# Designing of an open innovation model in science and technology parks

**DOI:** 10.1186/s13731-022-00203-w

**Published:** 2022-01-25

**Authors:** Pari Bayat, Mehry Daraei, Amin Rahimikia

**Affiliations:** Department of Educational Management, Islamic Azad University, Khorramabad, Iran

**Keywords:** Science and technology park, Open innovation model, Grounded theory, Factor analysis

## Abstract

The main purpose of this research is to design and test an open innovation model in science and technology parks through a mixed exploratory approach. The data collection tool in the qualitative section is a semi-structured interview with 15 experts in the field of science and technology parks. Purposive sampling was used until theoretical saturation is achieved. The grounded theory was used to coding the interviews. As a result of open, axial, and selective coding in MAXQDA, 1551 free codes, 202 concepts, 73 sub-codes, and 21 main codes extracted. These codes categorized in six themes of causal conditions, central phenomenon, strategies, contextual conditions, intervening conditions, and outcomes. Initial questionnaire was administrated among 516 experts and managers of science and technology parks in Tehran province and affiliated companies. Confirmatory factor analyses showed high model fit with CFI equal to .93 and high internal consistency for the total score equal to .98. The results of this study showed that the proposed model of open innovation in science and technology parks in Iran has a good fit.

**Supplementary Information:**

The online version contains supplementary material available at 10.1186/s13731-022-00203-w.

## Introduction

Historically, organizations developed new technology for their own products internally (Wyld, 2010) that led to closed innovation approaches with a restricted interactions with outside knowledge and technology. Gradually, industrial sections were encouraged to transfer external technologies to their internal knowledge to accelerate internal innovation (Beamish & Lupton, 2009). Along with these development, Henry Chesbrough invented the open innovation paradigm in 2003 for the first time. Open innovation is defined as the purposeful use of input knowledge with the aim of facilitating and accelerating internal innovation and utilizing output knowledge to expand it in society and use innovation (Chesbrough et al., 2006). Keupp and Gassmann (2009) also defined open innovation as the revelation and permeability of corporate organizational boundaries and the external environment.

Chesbrough, the founder of the open innovation paradigm, identifies some of the organizational capabilities needed to move toward open innovation as follows: (1) networking and network management; (2) knowledge management, intellectual management; (3) technology monitoring; (4) market knowledge; (5) predicting market needs, and (6) product and technology planning. The goal of open innovation is to capitalize on the discoveries and innovations of others in the innovation process, as opposed to closed processes, in which companies operate only with their own professional ideas, capabilities and capacities (Schwab et al., 2013).

In the following years, various theories about open innovation emerged. Tushman and Anderson (1986) proposed the theory of discontinuities innovation versus fundamental innovation. According to this theory, knowledge is the main foundation of any company's ability, then any change in knowledge indicates a change in the organization's ability to provide a new service and it emphasized the organization's ability to innovate. Martins and Terblanche (2003) in their theories expressed the cultural factors influencing organizational innovation as risk acceptance, open communication between employees, ambiguity and conflict, encouraging new theories, job commitment, strong and supportive leadership, customer orientation, and increasing benefits. Baumard (2009) expressed the theory of innovation with an integrated and networked system; its most important components are introduced as integrating development strategies between different internal and external organizations, simulation in research and development and close communication with people in the society, company flexibility to change and speed in development and more focus on quality to price (Spithoven et al., 2013).

There are many benefits to using open innovation, including early participation in new technologies and job opportunities, delays in financial commitment, mitigation of losses on initial outflows, and delays in outflows if investment is possible (Vanhaverbeke et al., 2008). Open innovation also makes it possible to exploit the knowledge of smart people outside the company; it enables the simultaneous use of domestic and foreign research and development; there is no need for the researcher to invest in order to make a profit from the research; the key to success will be the best use of internal and external ideas (Docherty, 2006). Mansfield (1986) showed that innovative projects that rely heavily on external development have shorter development times and require less investment than similar projects that rely entirely on R&D.

In this study, Grounded Theory (Strauss & Corbin, 1990) was used to develop the model for the open innovation in science and technology parks in which using a set of data, a theory is generated. In this approach, the data are analyzed through open, axial, and selective coding. Growing connections between the developed concepts merge to form an integrated framework with a single central category in the axial coding also called theoretical coding (Glaser, 1987). Strauss and Corbin (1990) recommend examining the data and codes using a coding paradigm that concentrates on and relates causal conditions, context, intervening conditions, strategies, and outcomes to figure out the relationships between the categories.

In Iran, in the field of open innovation development, extensive research has been done and its role in university–industry cooperation has been pointed out and research cooperation has shown a positive and significant effect on university–industry cooperation (Madhooshi & Kiakajuri, 2018). An open innovation model in small and medium enterprises has been designed using grounded theory (Babaei Farsani et al., 2018). In another study, the pattern of establishing open innovation in education is designed (Rashki et al., 2020). Despite extensive research in the field of open innovation in Iran, only one study has designed a model for open innovation in science and technology parks (Mirfakhredini et al., 2016) and no study has developed an instrument to measure the model. In this study, we designed, developed, and validated an instrument to measure the dimensions of the open innovation model in science and technology parks in Iran.

## Methods

In this research, the exploratory mixed method has been used. Research strategy, in the qualitative phase was grounded theory with the aim of theorizing. Grounded theory is a method for constructing a theory based on facts and data (Glaser & Strauss, 1967). The statistical population of the study includes all experts and activists in the field of science and technology parks and the sample size includes 15 of these people who were selected by purposive sampling. The inclusion criteria for participants were having at least 5 years experience of working at science and technology parks and a relevant university/college degree. Preliminary data were collected through semi-structured interviews with the target population. Data collection continued until theoretical saturation was reached. Data obtained from interviews were coded through grounded theory research approach and analyzed using MAXQDA software. The interviews were conducted on the phone due to the limitations regarding COVID-19 conditions that caused difficulties for in person interviews. Dimensions, main codes, and subcodes extracted from the interviews provided in Table 1.

**Table 1:** Dimensions, main codes, and sub-codes extracted from the interviews

Dimensions	Main codes	Sub-codes
Causal conditions	Cost and financial problems	Cost issues
		Financial barriers
	Lack of proper mechanisms to enforce the rules	Intellectual property issues
		Sharing criteria issues
		Sharing criteria issues
	Rapid changes in technologies and demands	Rapid technological changes
		Change the will of the people
Central phenomenon	The process of transferring knowledge and technology from outside to inside and vice versa	Identify new technology and ideas
		Outsourcing
		Buying technology
		Obtaining a license
		Technology sales
		Licensing
		Open source
		Reproductive companies
Strategies	Teamwork and collective thinking	Team working
		Group thinking
	Participation and cooperation	Academic elite participation
		Participation of community members
	Increase absorption capacity	Ability to identify external knowledge
		Ability to attract external knowledge
		Ability to adapt external knowledge
	Scientific and research interactions	Interact with domestic universities
		International interactions
	Exhibition of achievements	Conferences and seminars
		Exhibitions and festivals
	Creating an innovation network	Formal and informal communications
		Network information management
	Park management specialty	Technology management
		Financial management
		Performance management
		Marketing management
	Motivational factors	Internal motivation
		External motivation
Contextual conditions	Development of park infrastructure	Public service
		Patents
		Technical and specialized services
		Educational consulting services
		Credit facilities
	Provision and allocation of financial resources	Allocation of financial resources
		Facilities for attracting financial credit
		Venture capital
	Structural and content	Structural factors of the park
		Creating an open culture
		Park environment
	Development of human	Expert staff
		Experienced partners
		Competitors
		Partner customers
		Knowledge suppliers
		Financial investors
		Attract the elite
Intervening conditions	Weakness in determining the effectiveness of the park	Lack of sufficient transparency
		Complexity of park functions
		Uncertainty about resource efficiency
		Weakness in identifying value-added factors
		Lack of matching plans for parks
	Organizational constraints	Management factors
		Corporate conservatism
		Administrative bureaucracy
		Closed view of human resources
Outcomes	The growth of the knowledge-based economy	Commercialize ideas
		Commercialize university output
		Creating and quickly entering new markets
		Reducing costs and risk
		Increasing product quality
		Employment
	Strengthen innovative social activities	Increasing innovation
		Increasing the number of open innovators
		Strengthen the spirit of extroversion
	Improving cultural factors	Improving the culture of teamwork
		Increasing the trustability
		Expanding the partnership participation

In the quantitative phase, we used the developed questionnaire from the qualitative part to collect the quantitative data. The questionnaire is a self-reported 100-item with a five-point Likert scale and six factors of causal conditions, central phenomenon, strategies, contextual conditions, intervening conditions, and outcomes (see Additional file 1: Appendix S1). Data collection in quantitative phase was conducted virtually through digital format of the questionnaire sent to the participants’ email addresses. We tested the findings acquired from the qualitative part using confirmatory factor analyses using SPSS v24 and Mplus v7.4 in a convenient sample consisting 516 participants. Sample size was calculated using GPower v3.1.9.2 with the effect size of 0.35 and power (1-β) of 0.95.

## Findings

### Development and content validity of the questionnaire

In the qualitative phase of the research, open, axial and selective coding were used. In open coding process, the data obtained from the interviews were carefully reviewed, the main and sub-themes were identified. A total of 1551 free code, 202 sub-codes, 73 main codes, and 21 dimensions were extracted. Table 3 shows the dimensions, sub-codes, and main codes extracted from the interviews.

**Fig. 1:**
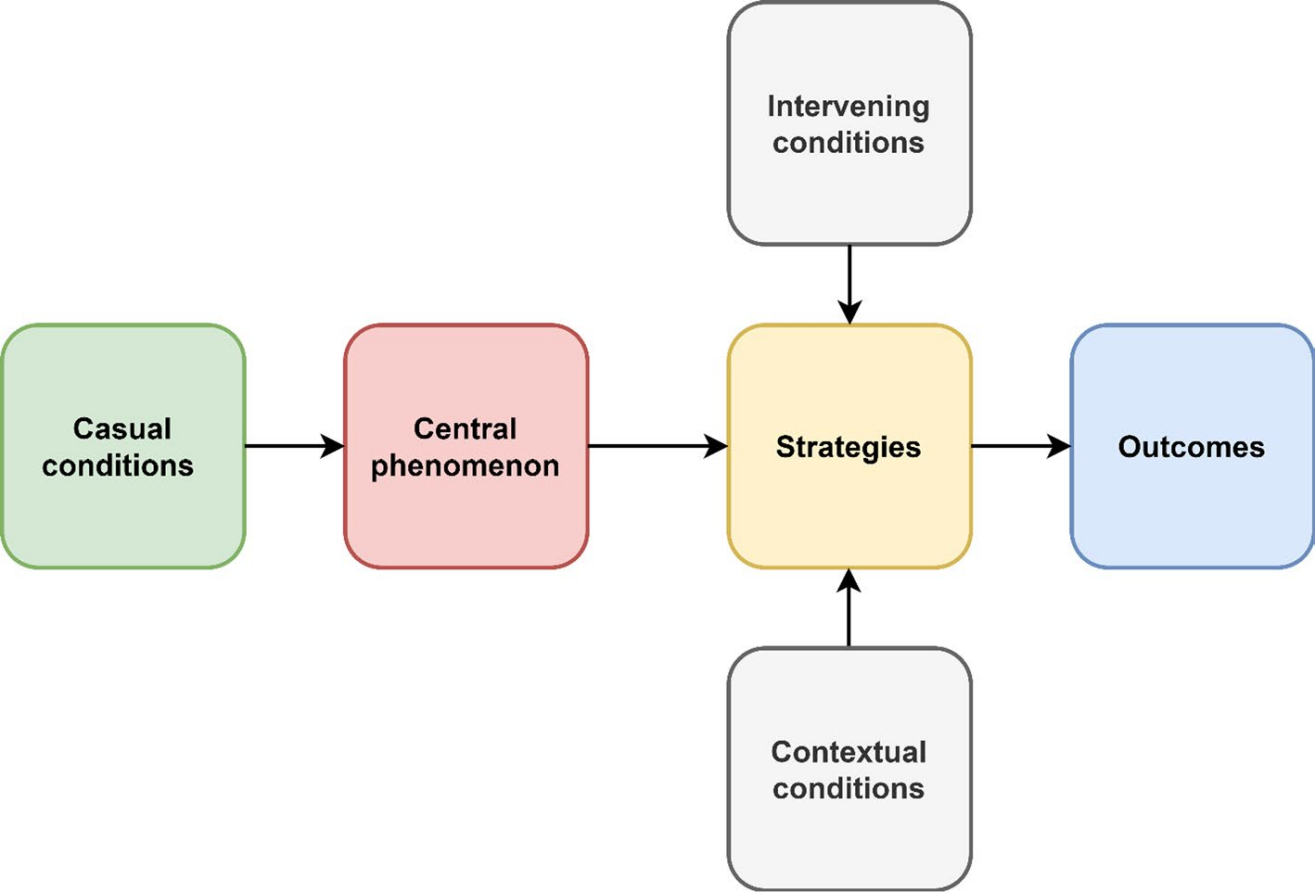
The model of the axial coding based on Grounded Theory (Corbin & Strauss, 2014)

In selective coding, the main variable or underlying process embedded in the data, how, its stages and consequences are plotted (Glaser & Strauss, 1967). Based on the obtained relationships, the concepts obtained from open and axial coding were linked to each other in the selective coding stage and were reflected as a model in science and technology parks according to Fig. 1. This figure displays the relationships between dimensions in the model and how the causal conditions was led to outcomes.

After developing the open innovation model, the initial items of the Open Innovation Questionnaire of Science and Technology Parks were written. The content validity of the questionnaire was examined by an expert panel. Content analysis was performed using content validity index (Waltz & Bausell, 1981) and 9 items were removed in this phase.

### Confirmatory factor analysis

The initial questionnaire was piloted and members of Tehran Science and Technology Park answered the questions of the questionnaire and the data were studied using confirmatory factor analyses. Kaiser–Meyer–Olkin test was performed to evaluate the adequacy of the research sample. The value obtained is equal to 0.979 which indicates that the sample size is sufficient, Table 2 shows these results.

**Table 2:** KMO and Bartlett test of sphericity

Kaiser–Meyer–Olkin sampling adequacy scale	0.979
Bartlett test of sphericity	
Chi-square	89,665.230
df	4950
*p*-value	0.000

Values above 0.70 indicate the adequacy of the sample size. The KMO value showed that the variance of the sample variables was sufficient for the structural validity test. In addition, the Bartlett test was significant at the level of *p* = 0.05. The Bartlett sphericity test measures the correlation between variables and investigates whether the variables are sufficiently correlated to perform structural validity. Therefore, the assumptions for conducting factor analysis were approved.

The matrix of components and items of the questionnaire after rotation showed that all items had a factor load greater than 0.5, so none of the items were removed (Truong & McColl, 2011). According to the developed model in qualitative phase, we tested the fit of the first- and second-order six-factor model solution. The results of confirmatory factor analysis in Mplus v7.4 with maximum likelihood estimation method and 20 iterations are reported in Table 3.

**Table 3:** Open innovation model fit for the first-order model

Value	Fit index
	Chi-square test of model fit
9819.180	Value
3778	Degrees of freedom
0.0000	*P*-value
	RMSEA (root mean square error of approximation)
0.056	Estimate
0.054–0.057	90 percent CI
0.934	CFI
0.913	TLI
0.063	SRMR (standardized root mean square residual)

The result of confirmatory factor analysis showed a comparative fit index equal to 0.934 which indicates a good fit (Kline, 2005) and the residual square root of the standardized root mean is 0.056. The RMSEA below 0.08 indicates the low error of the measurement model (Hair et al., 2010). The factor loadings of items in their respective factors for the first-order model are presented in supplementary files.

Second-order model solution was performed to compare it with the first-order model. This analysis also was performed using Mplus v7.4 and maximum likelihood estimation method. Table 4 shows the fit indices for the second-order model using confirmatory factor analysis.

**Table 4:** Open innovation model fit for the second-order model

Value	Fit index
	Chi-square test of model fit
16,116.369	Value
4697	Degrees of freedom
0.0000	*P-*value
	RMSEA (root mean square error of approximation)
0.069	Estimate
0.067–0.070	90 percent CI
0.875	CFI
0.868	TLI
0.066	SRMR (standardized root mean square residual)

The comparative fit index of the model was equal to 0.875, hence, the fit indices reduced compared to the first-order model. Since, the first-order model has a better fit it was determined as the final model of open innovation questionnaire.

### Reliability

Cronbach’s alpha was estimated as an index of reliability, it measures internal consistency of the items in their pertinent factors and indicates the correlation between items and the construct under study. Table 5 displays Cronbach's alpha values for open innovation factors and the total score.

**Table 5:** Cronbach’s alpha of the factors and total score

Factors	Cronbach’s alpha
Causal conditions	0.963
Central phenomenon	0.973
Strategies	0.978
Contextual conditions	0.986
Intervening conditions	0.971
Outcomes	0.984
Open innovation model	0.980

Cronbach's alpha of the factors varies between 0.96 and 0.98 and the Cronbach's alpha of the whole questionnaire was estimated 0.98, which indicates the high internal consistency of the factors and the total score. According to the results, the open innovation model questionnaire indicated to have sufficient validity and reliability for science and technology parks in Iran.

## Discussion

The aim of the present study was to design and test an open innovation model in Iranian science and technology parks, the mixed exploratory research approach. As a result of the qualitative part, six themes of causal conditions, central phenomenon, strategies, contextual conditions, intervening conditions, and outcomes were extracted and the initial questionnaire was developed. Psychometric properties of the questionnaire were studied; validity of the questionnaire was measured using confirmatory factor analysis and reliability was estimated through Cronbach’s alpha. The results showed that the open innovation questionnaire for science and technology parks is a valid and reliable instrument and may be helpful with finding plausible existing deficiencies and limitations for utilizing open innovation in designing, developing and improving of science and technology parks.

In the interviews conducted in this study, the importance and usefulness of using open innovation in science and technology parks in Iran was emphasized and the interviewees mentioned many benefits for it that were reduced to sub-codes, main codes, and themes. Consistent with the findings of this study, Spithoven et al. (2013) examined the impact of open innovation on the innovative performance of small and medium enterprises compared to large firms and found that the impact of open innovation in small and medium enterprises is often different. Crema et al. (2014) also analyzed the relationship between company strategy, open innovation, and innovation performance with a focus on small and medium-sized firms and concluded that firms that follow an innovative strategy invest more in main technical skills and competencies and companies that choose diversity strategies are more likely to choose open innovation management practices exclusively. A meta-analysis of the literature on open innovation in small and medium-sized enterprises showed that they improve their overall innovation performance by choosing open innovation. They found that a large number of studies were conducted with a quantitative approach. Surprisingly, unlike many other disciplines, North American researchers have made a limited contribution but European scientists, along with some researchers from Korea, China and developing countries, have been working actively in this field (Hossain & Kauranen, 2016).

Similar study on open innovation in cyber security research institutes through Grounded Theory approach resulted in 10 sub-categories and three main categories (Ghouchani Khorasani et al., 2019). Babaee Farsani et al. (2018) also designed an open innovation model in active small- and medium-sized enterprises using Grounded Theory led to 34 sub-categories and 11 main categories in which some sub-categories were similar to sub-categories founded in the current study such as “increase absorption capacity”, “improving cultural factors”, and “teamwork and collective thinking”. Hence, literature review showed applying Grounded Theory in model development for open innovation in industry has been functional and beneficial as it was for development of a model for open innovation in science and technology parks.

## Conclusions

In the interviews conducted in this research, the importance and usefulness of using open innovation in science and technology parks in Iran was emphasized and the interviewees mentioned many benefits for it. However, participants noted that limitations, such as the lack of technology appropriate to the organizational conditions in the market, being costly, and relatively time-consuming process for implementation that need to be addressed by the authorities. In sum, the results of the present study, which was conducted with the aim of development and validation an open innovation model in Iranian science and technology parks, showed that this model is suitable for implementation in Iranian context and can measure the open innovation in science and technology parks to be used by managers, and researchers.

## Implications for stakeholders

We recommend that each science and technology park assign to one specific industry since it facilitates for managers to design, develop and enhance the open innovation for that particular industry with regard to the characteristics of it. Establishing science and technology parks in universities could be helpful to quicker exchange of open innovation interactions between universities and parks. Last but not the least, the developed questionnaire in this study could be used as a tool to discover shortcomings and deficiencies in the path of promoting open innovation in the science and technology parks.

## Supplementary Information

**Additional file 1.** Open innovation questionnaire for science and technology parks.

## Data Availability

Raw data will be available upon request to the corresponding author.

## Abbreviations

R&D: Research and development
KMO: Kaiser–Meyer–Olkin
Df: Degree of freedom
RMSEA: Root mean square error of approximation
CFI: Comparative fit index
TLI: Tucker–Lewis index
SRMR: Standardized root mean square residual
