# Supplementary material for: Designing of an open innovation model in science and technology parks
Source: J Innov Entrep. 2022 Jan 25;11(1):4. doi: 10.1186/s13731-022-00203-w (PMC8787032; doi:10.1186/s13731-022-00203-w)
Supplement: Supplementary file 1 — Additional file 1. Open innovation questionnaire for science and technology parks. [file 13731_2022_203_MOESM1_ESM.docx]

Instructions: Please select the option that corresponds to the reality of open innovation in your science and technology parks.

Options: 1: completely disagree, 2: disagree, 3: not agree not disagree, 4: agree, 5: completely agree.

| **No.** | **Items** | **1** | **2** | **3** | **4** | **5** |
| --- | --- | --- | --- | --- | --- | --- |
| 1 | Low durability of start-ups limits the implementation of open innovation due to high costs of developing innovative ideas. |  |  |  |  |  |
| 2 | The inability of intellectual property as collateral for knowledge-based companies limits the implementation of open innovation. |  |  |  |  |  |
| 3 | Inadequate methods of earning money for research and technology funds limit the implementation of open innovation. |  |  |  |  |  |
| 4 | Loan-based models are detrimental to knowledge businesses in the implementation of open innovation. |  |  |  |  |  |
| 5 | One of the causal factors affecting the implementation of open innovation is the lack of appropriate mechanisms for intellectual property rights. |  |  |  |  |  |
| 6 | Familiarity of companies with the principles, rules and regulations of open innovation is one of the causal factors affecting its implementation. |  |  |  |  |  |
| 7 | One of the causal factors affecting the implementation of open innovation is the problems in the knowledge management system inside and outside the organization. |  |  |  |  |  |
| 8 | Rapid changes in existing technologies in society are one of the causal factors affecting the implementation of open innovation. |  |  |  |  |  |
| 9 | Growing awareness and changing the demands of the people of the society are among the causal factors influencing the implementation of open innovation. |  |  |  |  |  |
| 10 | Recognizing and searching for signs of technological change to identify new and up-to-date ideas in the world is one of the most important components for knowledge transfer. |  |  |  |  |  |
| 11 | Companies outsource their research to stay ahead. |  |  |  |  |  |
| 12 | Companies outsource their research to create development. |  |  |  |  |  |
| 13 | Companies outsource their research to increase productivity. |  |  |  |  |  |
| 14 | Companies outsource their research to reduce costs. |  |  |  |  |  |
| 15 | Companies outsource their research to reduce risk. |  |  |  |  |  |
| 16 | One way to transfer knowledge is to purchase intellectual property and patents from external sources. |  |  |  |  |  |
| 17 | One way to transfer knowledge is to get a license to exploit foreign technologies and ideas. |  |  |  |  |  |
| 18 | One way to transfer knowledge is to sell domestic patents to the market. |  |  |  |  |  |
| 19 | One way to transfer knowledge is to sell intellectual property to the market for greater profits. |  |  |  |  |  |
| 20 | Licensing the use of internal technologies to external partners is a way of knowledge transfer. |  |  |  |  |  |
| 21 | Introduction of internal technologies without regard to direct financial benefits in the short term is involved in knowledge transfer. |  |  |  |  |  |
| 22 | Creating new companies based on the internal knowledge of the parent company is effective in transferring knowledge. |  |  |  |  |  |
| 23 | One way to implement open innovation is to work as a team and jointly invest in companies. |  |  |  |  |  |
| 24 | Due to the rapid change in the working conditions of the company compared to the changes of individuals, collective thinking is of particular importance. |  |  |  |  |  |
| 25 | The cooperation and participation of innovators and elites has a significant impact on presenting products and services to the market. |  |  |  |  |  |
| 26 | Customer and consumer participation in market research plays an important role in discovering needs. |  |  |  |  |  |
| 27 | Reforms requested by customers and consumers lead to product development and change the company's performance. |  |  |  |  |  |
| 28 | The ability of companies to identify foreign knowledge has a significant impact on increasing the absorption capacity. |  |  |  |  |  |
| 29 | The ability of companies to attract foreign knowledge has a significant impact on increasing the absorption capacity. |  |  |  |  |  |
| 30 | The ability of companies to apply foreign knowledge has a significant impact on increasing the absorption capacity. |  |  |  |  |  |
| 31 | Scientific and research interaction of companies with domestic universities and educational centers as one of the strategies has a great impact on open innovation. |  |  |  |  |  |
| 32 | The scientific and research interaction of companies with domestic research and development institutions as one of the strategies in open innovation has a great impact. |  |  |  |  |  |
| 33 | Sharing the knowledge of commercializing ideas with international experts is one of the open innovation strategies. |  |  |  |  |  |
| 34 | Domestic and international conferences and seminars, while introducing the latest technologies in the world, cause companies to get acquainted with industry leaders. |  |  |  |  |  |
| 35 | Domestic and international conferences and seminars, while creating numerous new innovative opportunities, introduce companies to top competitors. |  |  |  |  |  |
| 36 | Domestic and international exhibitions and festivals introduce companies to the latest experiences and scientific achievements. |  |  |  |  |  |
| 37 | Innovative products and services are offered to other companies in domestic and international exhibitions and festivals. |  |  |  |  |  |
| 38 | Establishing strong formal or informal communication (based on trust) between members has important role in the improvement of innovation network. |  |  |  |  |  |
| 39 | The Memorandum of Understanding on joint cooperation of regional or international science parks is effective in creating an open innovation network. |  |  |  |  |  |
| 40 | Network information flow management as a strategy has a special place in the implementation of open innovation. |  |  |  |  |  |
| 41 | The expertise of senior managers in the field of marketing is one of the factors influencing the implementation of open innovation. |  |  |  |  |  |
| 42 | The expertise of senior managers in controlling and monitoring the performance of companies is one of the factors affecting the implementation of open innovation. |  |  |  |  |  |
| 43 | The expertise of senior managers in controlling and monitoring the performance of companies is one of the factors affecting the implementation of open innovation. |  |  |  |  |  |
| 44 | The level of interest and inner pleasure of innovation agents in increasing their level of knowledge and its transfer is effective on the implementation of open innovation. |  |  |  |  |  |
| 45 | The level of interest of innovation agents in increasing their knowledge and its transfer is effective on the implementation of open innovation. |  |  |  |  |  |
| 46 | Teaching introspective and extroverted skills to innovation agents is effective in implementing open innovation. |  |  |  |  |  |
| 47 | Introducing successful open innovation projects motivates innovators to implement open innovation. |  |  |  |  |  |
| 48 | Creating the right standard of living for employees and meeting their service and public needs is essential to implementing open innovation. |  |  |  |  |  |
| 49 | The existence of innovative infrastructure, patents and research for innovators in the parks is important for the implementation of open innovation. |  |  |  |  |  |
| 50 | Providing technical and specialized services for knowledge-based companies is an essential necessity for implementing innovation. |  |  |  |  |  |
| 51 | Providing consulting training services for knowledge-based companies is an essential necessity for implementing innovation. |  |  |  |  |  |
| 52 | Providing credit facilities for knowledge-based companies is an essential necessity for implementing innovation. |  |  |  |  |  |
| 53 | Examining financial issues and allocating financial resources are among the underlying factors of open innovation. |  |  |  |  |  |
| 54 | The provision of financial and credit facilities, such as collateral for companies, provides the basis for open innovation. |  |  |  |  |  |
| 55 | Creating risky financing institutions is a good platform for open innovation. |  |  |  |  |  |
| 56 | The degree of recognition as one of the structural factors of the park has an effect on the park's approach to open innovation. |  |  |  |  |  |
| 57 | The degree of focus as one of the structural factors of the park has an effect on the park's approach to open innovation. |  |  |  |  |  |
| 58 | The degree of complexity as one of the structural factors of the park has an impact on the park's approach to open innovation. |  |  |  |  |  |
| 59 | The culture of accepting the sharing of ideas by different people is the basis of open innovation. |  |  |  |  |  |
| 60 | The culture of opening the barriers between knowledge within the organization and the outside world is a predisposing factor to open innovation. |  |  |  |  |  |
| 61 | Creating a culture of trust between investors, partners, and the groundwork is a predisposing factor for open innovation. |  |  |  |  |  |
| 62 | Creating a culture of collaboration between open innovation actors paves the way for open innovation. |  |  |  |  |  |
| 63 | The adaptability of companies to the changes of society is one of the environmental factors affecting open innovation. |  |  |  |  |  |
| 64 | The flexibility of internal teams to transfer knowledge is one of the environmental factors affecting open innovation. |  |  |  |  |  |
| 65 | Having expert staff is important to pave the way for open innovation. |  |  |  |  |  |
| 66 | Having experienced partners is important to build credibility for investing in open innovation. |  |  |  |  |  |
| 67 | Examining competitors' proposals is important in the future market need to facilitate the open innovation. |  |  |  |  |  |
| 68 | Customer feedback is important as a partner in creating innovation to facilitate the open innovation. |  |  |  |  |  |
| 69 | The existence of universities as suppliers of knowledge and enrichment paves the way for open innovation. |  |  |  |  |  |
| 70 | Existence of research and development centers and innovation as suppliers of knowledge and enrichment is the basis of open innovation. |  |  |  |  |  |
| 71 | The existence of financial investors to provide financial resources is the basis for open innovation. |  |  |  |  |  |
| 72 | Attracting elite and creative people in the company to use new ideas in research is a predisposing factor for open innovation. |  |  |  |  |  |
| 73 | Lack of sufficient transparency in the park's mission, from the production chain of ideas to commercialization, hinders the implementation of open innovation. |  |  |  |  |  |
| 74 | Insufficient knowledge of the park's internal operations and processes prevents the implementation of open innovation. |  |  |  |  |  |
| 75 | Uncertainty about the effectiveness of the resources available in the park prevents the implementation of open innovation. |  |  |  |  |  |
| 76 | The lack of identification of value-added factors for companies due to their presence in the park prevents the implementation of open innovation. |  |  |  |  |  |
| 77 | Failure to develop a comprehensive plan for the implementation of parks hinders the implementation of open innovation. |  |  |  |  |  |
| 78 | Preventive effective policies are of important managerial factors in creating change towards innovation, which prevents the implementation of innovation. |  |  |  |  |  |
| 79 | Excessive conservatism of companies in transferring knowledge prevents the implementation of open innovation. |  |  |  |  |  |
| 80 | Fear of competitors' copying of innovative ideas prevents open innovation. |  |  |  |  |  |
| 81 | Administrative bureaucracy prevents companies from implementing open innovation. |  |  |  |  |  |
| 82 | The unwillingness of employees within the organization to communicate openly with outside the organization limits the implementation of open innovation. |  |  |  |  |  |
| 83 | Closed views and misconceptions of employees about teamwork limit the implementation of open innovation. |  |  |  |  |  |
| 84 | Weaknesses in the culture of cooperation between firm employees and innovation partners limit the implementation of open innovation. |  |  |  |  |  |
| 85 | By implementing open innovation, the sales of the company's internal ideas and technologies to the market will increase. |  |  |  |  |  |
| 86 | Creating revenue with the growth of a knowledge-based economy is one of the benefits of implementing innovation. |  |  |  |  |  |
| 87 | Creating business opportunities from untapped domestic research is one of the benefits of open innovation. |  |  |  |  |  |
| 88 | Commercializing the output of universities and research centers is one of the benefits of implementing innovation. |  |  |  |  |  |
| 89 | Creating a new market for new products and entering them quickly is one of the economic outcomes of implementing innovation. |  |  |  |  |  |
| 90 | By implementing open innovation, companies' costs and risks are reduced through joint ventures and increased product quality. |  |  |  |  |  |
| 91 | Increasing product quality based on the opinion of the community is one of the benefits of implementing innovation. |  |  |  |  |  |
| 92 | Implementing open innovation, increasing diversity and the number of jobs by creating new products and services will reduce the unemployment of the educated individuals. |  |  |  |  |  |
| 93 | With the implementation of open innovation, inventions, new ideas and innovative products increase. |  |  |  |  |  |
| 94 | With the implementation of open innovation, the number of open innovators increases. |  |  |  |  |  |
| 95 | Creating open thinking in accepting new ideas increases with the implementation of open innovation. |  |  |  |  |  |
| 96 | The spirit of extroversion and attention to the environment increases with the implementation of open innovation. |  |  |  |  |  |
| 97 | By implementing open innovation, dynamic, empowering, enthusiastic, and motivated teamwork will be formed. |  |  |  |  |  |
| 98 | With the implementation of open innovation, scientific synergy, collectivism, and consensus are achieved. |  |  |  |  |  |
| 99 | By implementing open innovation, employees' trust in each other at different levels leads to increased scientific interactions. |  |  |  |  |  |
| 100 | Expanding the culture of stakeholder participation and cooperation is one of the cultural outcomes of open innovation. |  |  |  |  |  |
